# More than medications: a patient-centered assessment of Parkinson’s disease care needs during hospitalization

**DOI:** 10.3389/fnagi.2023.1255428

**Published:** 2023-09-28

**Authors:** Jessica Shurer, Shannon L. S. Golden, Paul Mihas, Nina Browner

**Affiliations:** ^1^CurePSP, Inc., New York, NY, United States; ^2^Goldsmith Research Group, Winston-Salem, NC, United States; ^3^Odum Institute for Research in Social Science, University of North Carolina at Chapel Hill, Chapel Hill, NC, United States; ^4^Department of Neurology, University of North Carolina at Chapel Hill, Chapel Hill, NC, United States

**Keywords:** Parkinson’s disease, hospitalization, patient-centered care, qualitative methods, focus groups

## Abstract

**Background:**

Parkinson’s disease (PD) increases the risk of hospitalization and complications while in the hospital. Patient-centered care emphasizes active participation of patients in decision-making and has been found to improve satisfaction with care. Engaging in discussion and capturing hospitalization experience of a person with PD (PwP) and their family care partner (CP) is a critical step toward the development of quality improvement initiatives tailored to the unique hospitalization needs of PD population.

**Objectives:**

This qualitative study aimed to identify the challenges and opportunities for PD patient-centered care in hospital setting.

**Methods:**

Focus groups were held with PwPs and CPs to capture first-hand perspectives and generate consensus themes on PD care during hospitalization. A semi-structured guide for focus group discussions included questions about inpatient experiences and interactions with the health system and the clinical team. The data were analyzed using inductive thematic analysis.

**Results:**

A total of 12 PwPs and 13 CPs participated in seven focus groups. Participants were 52% female and 28% non-white; 84% discussed unplanned hospitalizations. This paper focuses on two specific categories that emerged from the data analysis. The first category explored the impact of PD diagnosis on the hospital experience, specifically during planned and unplanned hospitalizations. The second category delves into the unique needs of PwPs and CPs during hospitalization, which included the importance of proper PD medication management, the need for improved hospital ambulation protocols, and the creation of disability informed hospital environment specific for PD.

**Conclusion:**

PD diagnosis impacts the care experience, regardless of the reason for hospitalization. While provision of PD medications was a challenge during hospitalization, participants also desired flexibility in ambulation protocols and an environment that accommodated their disability. These findings highlight the importance of integrating the perspectives of PwPs and CPs when targeting patient-centered interventions to improve hospital experiences and outcomes.

## Introduction

People with a clinical diagnosis of Parkinson’s disease (PD) experience more frequent and prolonged hospitalizations than their age-matched peers (Aminoff et al., 2011; Chou et al., 2011; Hobson et al., 2012; Kowal et al., 2013; Shahgholi et al., 2017; Su et al., 2018). Most hospitalizations occur in general wards and result from a comorbid disorder or health crisis, such as respiratory and urinary tract infections, cardiovascular diseases, falls, and fractures (Woodford and Walker, 2005; Braga et al., 2014; Lubomski et al., 2015; Gil-Prieto et al., 2016; Okunoye et al., 2020; Réa-Neto et al., 2021). It is well documented that during hospitalizations, person with PD (PwP) is at a higher risk of complications, including falls, medication errors, development of delirium and psychosis, and overall decline of their pre-existing motor and non-motor symptoms of PD (Derry et al., 2010; Gerlach et al., 2013; Lubomski et al., 2015; Skelly et al., 2017; Magnuszewski et al., 2022). Improved medication adherence during hospital stays, e-alerts to PD specialists upon admission, development of the Parkinson’s Foundation Aware in Care Hospital Kit, and recommendations for ward certification programs are among the calls for action and quality improvement interventions that have targeted hospital outcomes for PD (Azmi et al., 2019, 2020; Hobson et al., 2019; Aslam et al., 2020; Nance et al., 2020; Parkinson’s Foundation Hospital Safety Kits, 2003). However, to date, only a few studies have reported significant decreases in the length of hospital stay or complications during hospitalization for PD (Skelly et al., 2014; Azmi et al., 2020).

In the context of inpatient hospitalization for older adults and those with chronic and serious medical conditions, patient-centered care (PCC) has revealed benefits in intermediate and distal outcomes, and almost all studies have found positive relationships between PCC approaches and patient satisfaction (Counsell et al., 2000; Wolf et al., 2008; Rocco et al., 2011; Rathert et al., 2013). PCC places patients at the center of the healthcare decision-making process and recognizes the importance of their individual preferences and goals (Berwick, 2009; Institute of Medicine, 2014). Growing awareness of PCC delivery has resulted in the establishment of specialized multidisciplinary teams as the gold standard of outpatient care for PD, as well as the increasing application of palliative care, a traditionally team-based model of care, for the management of the physical, emotional, and spiritual needs of PwPs (Eggers et al., 2018; Connor et al., 2019; Vlaanderen et al., 2019; Bhidayasiri et al., 2020; Kluger et al., 2020; Rajan et al., 2020; Lennaerts-Kats et al., 2022).

Presently, the voices of PwPs and their care partners (CPs) regarding their experiences during hospitalization are not well represented in the literature, and most research on PCC for PD has focused on outpatient care. Studies that qualitatively investigated hospitalization for PD primarily highlighted medication mismanagement, struggles with postoperative confusion, and deterioration of motor symptoms; however, they minimally captured patient-reported needs or experiences of CPs during hospitalization (Barber et al., 2001; Buetow et al., 2009; Gerlach et al., 2012; Carney Anderson and Fagerlund, 2013; Read et al., 2019).

By applying open–ended questions qualitative methods gather detailed and nuanced accounts of participants’ experiences, perceptions, and behavior. Compared to quantitative methods, which are intended to achieve the breadth of understanding of a topic, qualitative methods dive deep into individual experiences and the context surrounding them (Patton, 2002).

Unlike quantitative research, which emphasizes data generalization by employing sample size calculation and randomization, qualitative methods place primary emphasis on saturation, which means collecting the information until no new substantive insight emerges (Francis et al., 2010). To achieve saturation, researchers often use purposeful sampling by recruiting individuals who are especially knowledgeable about or experienced with a phenomenon of interest, available and willing to participate, and can communicate experiences and opinions in an articulate, expressive, and reflective manner (Guest et al., 2006). This approach can collect robust and meaningful data per participant, and thus fewer participants within the sample are needed to achieve saturation or “information power” of the sample (Malterud et al., 2016). The choice for qualitative data analysis is dictated by qualitative study methods and the research question. Since open–ended surveys, focus groups, and one on one interviews create information-rich and nuanced datasets, thematic analysis is commonly applied to this qualitative method. This involves creating codes (labels) to organize and describe the data, and then actively synthesizing the data by framing, interpreting, and/or connecting data elements to construct the themes (Kiger and Varpio, 2020). The advantage of thematic analysis is that it offers researchers flexibility concerning the type of research questions it can address, however, it also implies a systematic and iterative process that requires careful attention and interpretation of the data, and then a rigorous approach to identifying and validating themes (Braun and Clarke, 2006, 2012; Kiger and Varpio, 2020).

To expand the scope of patient-centered PD care from outpatient to inpatient settings, we employed qualitative research to gather and analyze valuable self-reported experiences of PwPs and CPs regarding hospitalization.

## Methods

### Study design

Focus groups were selected as the optimal methodology to capture first-hand experiences of PwPs and CPs, and to generate themes on PD care during hospitalization (Patton, 2002; Busetto et al., 2020). Participants either had a neurologist-confirmed clinical diagnosis of PD or were family members of a person with a neurologist-confirmed clinical diagnosis of PD and were able to participate in the interview. Hospitalization was defined as a planned (e.g., scheduled surgery or procedure) or unplanned (e.g., emergent or urgent admission) hospital stay for at least 24 h between January 2018 and July 2022. Patients hospitalized for deep brain stimulation surgery were excluded. All participants had to be at least 18 years old, with no upper age limit.

This study was conducted in accordance with the Declaration of Helsinki and was approved and overseen by the Office of Human Research Ethics the University of North Carolina at Chapel Hill. The participants provided verbal informed consent prior to any study activity including data collection. This study was supported by a Parkinson’s Foundation Community Outreach Resource Education grant.

### Data collection

Participants were recruited through clinician referrals, announcements shared with North Carolina-based PD support groups, and flyers placed at the outpatient neurology clinic at the University of North Carolina at Chapel Hill. A purposive sampling strategy was used to recruit a variety of PwPs and CPs with a range of hospital experiences (Table 1). The focus groups were capped at a maximum of five participants to allow for adequate time for each person to actively participate in the focus group discussion and accommodate for inherent challenges with communication and processing speed in PD. Quotas were applied to the patient sample to ensure a diverse representation, including demographic (e.g., sex, age, race) and clinical characteristics (e.g., stage of condition as defined by Hoehn and Yahr score, planned vs. unplanned hospitalization experiences) (Goetz et al., 2004). Half-way through recruitment, additional efforts were made to enroll participants from underrepresented demographics of the study.

**Table 1:** Demographic and clinical characteristics of the participants.

Demographics	Total PwP (*n* = 13)	Total CPs (*n* = 12)	Total (*n* = 25)
**Age *n* (%)**			
Below 55 years old	0	1 (8%)	1 (4%)
55–64 years old	0	1 (8%)	1 (4%)
65–74 years old	9 (69%)	2 (17%)	11 (44%)
75–84 years old	3 (23%)	7 (58%)	10 (40%)
Over 85 years old	1 (7%)	1 (8%)	2 (8%)
**Gender *n* (%)**			
Male	9 (69%)	3 (25%)	12 (48%)
Female	4 (31%)	9 (75%)	13 (52%)
**Highest level of education *n* (%)**			
High school	0	0	0
Some college	3 (23%)	1 (8%)	4 (16%)
Associate degree	4 (31%)	0	4 (16%)
Bachelor’s degree	1 (8%)	6 (50%)	7 (28%)
Master’s degree	2 (15%)	1 (8%)	3 (12%)
PhD	3 (23%)	4 (33%)	7 (28%)
**Race *n* (%)**			
White	10 (77%)	8 (67%)	18 (72%)
Black/African American	1 (8%)	1 (8%)	2 (8%)
Asian	2 (15%)	2 (17%)	4 (16%)
Multi-racial	0	1 (8%)	1 (4%)
**Yrs since diagnosis of PD *n* (%)**			
≥15 yrs	1 (8%)	3 (25%)	4 (16%)
11–14	5 (38%)	4 (33%)	9 (36%)
6–10	4 (31%)	3 (25%)	7 (28%)
3–5	3 (23%)	2 (17%)	5 (20%)
Less than 3 yr.	0	0	0
**H&Y stage *n* (%)**			
Stage 1	0	n/a	0
Stage 2	3 (23%)	n/a	3 (23%)
Stage 3	6 (46%)	n/a	6 (46%)
Stage 4	4 (31%)	n/a	4 (31%)
**Hospitalization type *n* (%)**			
Planned hospitalization	4 (31%)	0	4 (16%)
Unplanned hospitalization	9 (69%)	12 (100%)	21 (84%)

A semi-structured discussion guide, developed *de novo* by the research team based on a review of the published literature on hospitalizations in PD, was used to structure the focus groups (Supplementary Appendix S1). As the data collection progressed, the discussion guide was adapted to incorporate new issues raised by the participants. The questions focusing on aspects of hospital admission, inpatient experiences, discharge processes, interactions with the health system and team, and lived experiences of PwPs and CPs thought to be most relevant to patient- and family-centered outcomes. An experienced group moderator (J.S.) used probing questions to further expand the discussion. Prior to the focus groups, all the participants completed a brief questionnaire to capture their demographic information.

All focus groups were conducted virtually on the Zoom platform and were recorded and transcribed verbatim. Each group was 120 min in length. Identifiers were stripped from the transcripts, which were reviewed for accuracy. Participants received $20 honoraria. After conducting 7 focus groups, the research team determined that information power was achieved, and recruitment ended (Patton, 2002).

### Data management and analysis

Transcripts were independently reviewed by a multidisciplinary team of three researchers, including a movement disorders specialist (N.B.), a clinical social worker (J.S.), and a qualitative methods expert (S.G.), to identify emerging concepts related to hospital experiences. During the first phase of analysis, two investigators (N.B. and S.G.) independently read 3 transcripts before convening to define initial topics/concepts and develop a preliminary codebook. Coded data and transcripts were maintained in an electronic database, MAXQDA 2020 (VERBI software, 2019). An inductive thematic approach was used for analysis. The respective coded transcripts were compared during face-to-face meetings (N.B. and S.G.) to assess similarities and discrepancies regarding code names and code application. Based on these consensus meetings, researchers developed a final codebook that was systematically applied to the remaining transcripts. The team continued to review and code transcripts independently, meeting regularly to collaboratively discuss coding decisions and to resolve any coding differences through consensus. All coded transcripts were then reviewed by a third researcher (J.S.) to ensure consistency (Busetto et al., 2020). The varied perspectives of team members yielded a nuanced and robust interpretation of the results and all discrepancies among analysts were resolved. The research team (N.B., J.S., P.M.) analyzed each code and assessed conceptual relationships among them to develop higher-level categories and the relevant sub-themes within each category (Table 2). The findings were then condensed, and conclusions drawn.

**Table 2:** *A priori* codes, sub themes, themes and categories.

*A priori* codes	Sub themes	Themes	Categories
Declaring PD diagnosis/establishing PD identity		Importance of active acknowledgment of PD diagnosis by the health care team	The impact of PD diagnosis on the hospital experience and perception of care among PwP and CPs
Knowledge by the team about PD			
Attributes of Health Care Team			
Care partner advocacy			
Care partner experience			
Advance care planning	Preparedness for hospital admission	Different experiences with care during planned and unplanned hospitalizations	
Decision making			
PD comorbidities			
Packing to go to hospital			
Knowledge by the Health Care Team about PD	Communications with health care team before admission/at ER/after admission/on discharge		
Attributes of Health Care Team			
ER experience			
Patient experience			
Care partner advocacy			
Care partner experience			
Emotions about hospital stay	Care during hospital stay		
PD medications			
Hospital environment			
Patient experience			
Care partner advocacy			
Care partner experience			
Knowledge by the Health Care Team about PD			
Rehabilitation/Ambulation	Rehabilitation/ambulation during hospital stay		
Discharge instructions			
Knowledge by the Health Care Team about PD			
Attributes of Health Care Team			
Patient experience			
Care partner advocacy			
Care partner experience			
Emotions about hospital stay	Inconsistent availability of PD medications Delays in medication schedule Substitution of medications	Dissatisfaction with management of PD medications	The unique needs of PwP and CP during hospitalizations
PD medications			
Patient experience			
Care partner advocacy			
Care partner experience			
Knowledge by the Health Care Team about PD			
Care partner advocacy	Self-administration of medications		
Declaring PD diagnosis/establishing PD identity			
Patient experience			
Knowledge by the Health Care Team about PD			
Rehabilitation/Ambulation	Allied health clinician evaluations Encouragement of safe ambulation	Desire for flexibility in hospital protocols regarding falls risk and ambulation	
Hospital environment			
Patient experience			
Care partner advocacy			
Care partner experience			
Declaring PD diagnosis/establishing PD identity	Individualized assessment of falls risks		
Emotions about hospital stay			
Care partner advocacy			
Hospital environment	Preservation of independence in the hospital environment	The need for disability-informed hospital environment	
Discharge instruction			
Rehabilitation/Ambulation			
Declaring PD diagnosis/establishing PD identity			
Patient experience			
Care partner advocacy			
Emotions about hospital stay	Desire for accommodations for PD—specific care needs		
Declaring PD diagnosis/establishing PD identity			
Care partner advocacy			
Care partner experience			

## Results

### Participants

Seven focus groups were conducted. Participants included 12 PwPs (69% in the 65–74 age range and 28% non-white) and 13 CPs, including five PwP-CP dyads, for a total of 25 participants (Table 1). 84% of participants had unplanned hospitalizations and 64% of participants had lived with the PD diagnosis between 6 and 14 years at the time of hospitalization. All CPs reported unplanned hospitalizations of their loved one with PD. Ten participants were hospitalized at academic medical centers. During recruitment, participants were identified by their roles in the healthcare system (patient vs. family CP). Initially, the researches planned to create homogenic focus groups that thought would facilitate open discussion (Kaiser, 2009). However, during the recruitment, several PwPs in more advanced diseases stages expressed a preference for their CPs to be present during focus group and help navigate challenges with speech or slower processing speed, which made it difficult for them to fully participate in the discussion. In these PwP – CP dyads, the CP commonly participated in discussion by either voicing their own opinion about hospitalization or helping the PwP express their thoughts, thus playing the role of “patient’s voice.” The study included two focus groups consisting solely of CPs, one focus group with only PwPs, and the remaining four focus groups had a mix of participants (Table 3).

**Table 3:** Composition of the focus groups.

Focus group #	Subject ID	Category of participant	Planned or unplanned hospitalization	Academic or community hospital admission	Gender	Race/ethnicity
1	P_1_G_1_CP*	Care partner	Unplanned	Community	F	Multi – racial
1	P_2_G_1_PwP*	Patient	Unplanned	Community	M	White
1	P_3_G_1_CP*	Care partner	Unplanned	Community	F	White
1	P_4_G_1_PwP*	Patient	Unplanned	Community	M	White
1	P_5_G_1_PwP	Patient	Planned	Academic	F	White
2	P_6_G_2_PwP	Patient	Unplanned	Community	M	White
2	P_7_G_2_PwP	Patient	Planned	Academic	M	White
2	P_8_G_2_PwP*	Patient	Unplanned	Community	F	White
2	P_9_G_2_CP*	Care partner	Unplanned	Community	M	White
3	P_10_G_3_CP	Care partner	Unplanned	Academic	F	White
3	P_11_G_3_CP	Care partner	Unplanned	Community	F	White
3	P_12_G_3_CP	Care partner	Unplanned	Community	M	Asian
3	P_13_G_3_CP	Care partner	Unplanned	Community	M	Asian
3	P_14_G_3_CP	Care partner	Unplanned	Academic	F	White
4	P_15_G_4_PwP	Patient	Planned	Academic	F	White
4	P_16_G_4_PwP	Patient	Unplanned	Community	M	Asian
5	P_17_G_5_CP	Care partner	Unplanned	Academic	F	White
5	P_18_G_5_CP	Care partner	Unplanned	Academic	F	White
6	P_19_G_6_PwP	Patient	Unplanned	Academic	M	Asian
6	P_20_G_6_PwP*	Patient	Unplanned	Community	M	African American
6	P_21_G_6_CP*	Care partner	Unplanned	Community	F	African American
6	P_22_G_6_PwP	Patient	Planned	Academic	F	White
7	P_23_G_7_PwP*	Patient	Unplanned	Academic	M	White
7	P_24_G_7_CP*	Care partner	Unplanned	Academic	F	White
7	P_25_G_7_PwP	Patient	Unplanned	Community	M	White

Dyads of PwP - CP are marked with*.

### Resulting categories

The focus group discussions revealed rich descriptive and thematic data, however, this paper focuses on two specific categories: the impact of the PD diagnosis on the patient and family’s hospital experiences and perceptions of care, and the emergence of distinctive needs of PwPs and CPs during hospitalization.

Tables 4, 5 present the sub-themes and themes accompanied by the focus group participants’ representative quotes. Each quote is marked with participant number (P#), focus group number (G#), and whether the participant identified as PwP or CP.

In the following section, we outline the major themes of each category.

1. **Pre-existing PD diagnosis affected participants’ hospital experience and perception of care: “They acknowledged [PD] immediately… that was great!”**
**1.1. Acknowledgment of PD diagnosis by the health care team was important to participants.**

**Table 4:** The impact of PD diagnosis on the hospital experience and perception of care among PwP and CPs with representative quotes.

Themes/sub themes	PwP and CPs representative quotes
Importance of active acknowledgment of PD diagnosis by the health care team	The doctor convinced me that they knew what they are doing with Parkinson’s as well. So that was reassuring, even though most of the focus was on the hip. I did not have to tell [about Parkinson’s diagnosis] anesthesiologist or any of them. They were all aware. And as a matter of fact, he said to me, “Do not worry about it. We’ve got that under control and we are taking care of you.” So he was great. I wanted to make sure that there was somebody that could do a good job with hip replacement, but they have had patients that had Parkinson’s, so they knew that that was an element that was different. *(P15G4PwP)* The anesthesiologist was really good. He acknowledged Parkinson’s disease diagnosis immediately before I went into the surgery. *(P20G6PwP)* I think there’s a big educational deficit with a lot of providers, cause in a lot of instances, you tell them that you have Parkinson’s, and they get that deer-in-the-headlights look and they do not quite know how it impacts what it is they are looking at. Or, really, what it is. *(P25G7PwP)* I would end up being the one who bring [Parkinson’s diagnosis] up more than anybody [because] the medication [timing] was the issue. It wasn’t available in the pharmacy. So then of course now that starts throwing me off on timing wise. I do not think there was awareness of how important timing is of the Parkinson’s pills. *(P7G2PwP)* …they were not really that familiar with how it impacted this kind of a situation… The nurse did not ask me, “How often does he have to have the carbidopa?” or “How many pills is he on?” They did not ask anything about that and I felt that they should have. The people taking care of him really had no experience with Parkinson’s, and I felt that they needed serious retraining about Parkinson’s*. (P1G1CP)* I expected them to—whoever was treating him—to understand what Parkinson’s meant. It meant tremors, it meant that he had to take L-dopa on time… and I’m not supposed to sleep there next to him the whole time. So, I expected them to say, “Oh, well we understand, and we will make sure that he gets the proper treatment.” That’s what I thought. I did not understand that the people taking care of him really had no experience with Parkinson’s. I could not trust them. *(P24G7CP)*
**Different experiences with care during planned and unplanned hospitalizations**	
Planned hospitalization	I think I went and had opinions from three or four doctors, and I wanted to specifically have somebody that can convince me they knew what they had dealt with Parkinson’s patients. That was really important to me. [F, hip replacement] *(P15G4PwP)* That was all very organized… They had all my paperwork ready. I had my physical before the surgery and had a chat with the doctor, what they are gonna do and how long it’s gonna take and what I’m gonna need and how long I’ll be in the hospital. And so, it went very smoothly actually, and a lot of the paperwork I had done ahead of time. After my spine surgery, they were very encouraging, to get up and move and walk around as much as you can. They do not want you to stay in bed. [F, spine surgery] *(P22G6PwP)* I was prepared. Number one, I wasn’t alone. My daughter went with me. We had my list of medications and when I take them and how much I take. And even though they told me not to bring them, I brought my medications with me just in case I panicked and did not have them. I was evaluated by physical therapy before discharge, and it was very encouraging to know that I am safe to go home. And to have specific instructions for my knee rehabilitation. [F, knee replacement] *(P5G1PwP)* For my discharge instructions I left there [hospital] … and nobody got in touch with my neurologist—I had to make my appointments by myself. [M, cardiac ablation] *(P7G2PwP)*
Unplanned hospitalization	I had a hard time breathing and went to ER. I mentioned I had Parkinson’s disease when it was time to take my medication. I said that I brought medication, but they insisted that they draw it from their pharmacy rather than use my medication. My medication was delayed and eventually I took my own. [M, pneumonia] *(P16G4PwP)* In ER, I had to describe exactly what Parkinson’s is because they’ll look at my hand shaking… They just kind of look…I kind of had to explain to them and then sometimes they would ask more questions about what exactly the disease is. My medications were always late, but I did not bother addressing it. I was never there long enough to make it worthwhile to change. And all the focus was on my urinary tract infection. [F, urinary tract infection] *(P8G2PwP*) The whole thing [admission] is so hazy. You do not know whether you are being discharged or not, and you do whatever they tell you to do. You’re almost sleepwalking, you know? I could tell that some of the nurses were not as cognizant of what Parkinson’s really means and the implications of it. [M, pneumonia] *(P19G6PwP)* I brought my own walker. The moment I felt better, I started to move around and out of the bed. I did not want to lose ground. And I did not have physical therapy for a while. I think they came only when it was time to discharge me. My wife helped me with walking. [M, urinary tract infection/sepsis] *(P6G2PwP)* I do not remember physical therapy coming into the hospital at all. And I stayed in bed all the time. It’s what they wanted me to do. They were afraid I would fall. [F, urinary tract infection] *(P8G2PwP)* EMT took him to ER but would not let me ride with them. I said to EMT that he may be having a stroke, but ER nurses did not understand that. It was poor communication between the people who were there and the people who were delivering him. I wish that I had been in the ambulance because I would have known how it was said and how he usually looks. I would’ve been able to advocate for him immediately and say, “This is an emergency. I think he maybe had a stroke. His speech is impaired.” But that did not happen. I do not know what they thought he was there for. [M, stroke] *(P17G5CP)* I remember that we were waiting in the ER hallway, and he was extremely agitated. There were six people trying to hold him down and they could not. I thought it was due to the fact that his medication wasn’t working so I was giving him his medication. And I remember that one of the doctors got so mad at me that he took the glass of water from me so that [PwP] could not swallow his medication. But anyway, I gave him the medication and he was less agitated. [M, urinary tract infection] *(P18G5CP)* We went to ER probably 2:30–3:00 p.m. Oh and thank goodness I brought some of the pills with me “cause of course we got into the 5:00 pm and then 9:00 pm carbidopa/levodopa dose before he was in a room.” And the people in the ER were not willing to do any of that before he got upstairs. So, I gave him his medications in ER. [M, abdominal infection] *(P3G1CP)* Every time you go to a hospital, and you come home from the hospital, there’s like 30 doctor visits you have to schedule. You gotta go see a neurologist, you gotta see your GP, you gotta go see your cardiologist, your pulmonologist, and then rehab on top of that. And physically moving her [PwP] around can be difficult for her and for us. And so sometimes it was like we could not even contemplate her having the ability to do all of that. So, with discharge, it wasn’t just sometimes what can we do for her at home, but how are we gonna do all of this when she does not even have the strength. [F, pneumonia] *(P12G3CP)*

PwP gender and diagnosis of hospitalizations presented in parenthesis.

**Table 5:** The unique needs of PwP and CP during hospitalizations with representative quotes.

Themes/sub-themes	PwP and CPs representative quotes
**Dissatisfaction with management of PD medications**	
Inconsistent availability of PD medications	The medication was the issue. I brought my medication with me, they insisted that I could not have my own little stash. I needed to go to the pharmacy. And they took down whatever I was supposed to take. So okay, I take the pills that I’ve got with me, give them to the nurse who puts them down in the pharmacy and then I have to get them brought back up to me. And all of this is taking time that is pushing me off my normal schedule. *(P15G4PwP)* They said that the hospital did not have it [carbidopa]. They said, “Well, we’ll have to see if the pharmacy can get it.” And so I offered to bring it and they did not want me to bring it. It was after the third day that they finally got it straightened out, so I do not think he had carbidopa until just before time to leave. *(P21G6CP)*
Delays in medication schedule	Every 4 h, that has to be taken; otherwise, my reaction is awful. Leg starts kicking, the hands start kicking, the body shakes. So, I mean, it’s really an awful feeling. *(P19G6PwP)* I can tell when I need to take my medications. That’s when my tremors start when I’m not on time. I take it three times a day. As it wears off, I take it. I asked for it, it took them an hour to get it*. (P8G2PwP)* But I would also say when someone with Parkinson’s is a patient in the hospital, they are probably under stress due to health issues. They probably need their medication early some of the time, not just on time. *(P14G3CP)* Our biggest problem was the timing. They could not get the idea that the carbidopa/levodopa had to be given at a certain time particularly in relation to meals*. (P3G1CP)* We know they are hospital and they are not gonna be on the dime at the exact timing, so you have to be somewhat flexible. But with Parkinson’s patients, especially with someone who’s taking medication every 2 h, more than 15 min is very impactful. *(P12G3CP)*
Substitution of medications	They were giving me different looking medications that they assured me were the same thing, just a different manufacturer or whatever it was. Every time they gave me something and I looked at it and questioned it. I was only there for 2 days so it wasn’t an extended time period, but it was very disconcerting for me. *(P5G1PwP)*
Self-administration of medications	Now, what happened was that they did not have something I needed—I wanna say some ropinirole—they did not have it in stock. So that was a problem. And I quite honestly had my son and my husband go home and they sort of snuck it in… *(P15G4PwP)* I let my wife know to bring the Neupro patches. It would’ve been a couple days before it got to the pharmacy, so we just went with mine and I thought that was good cause they normally do not allow you to do that. *(P25G7PwP)* And so I tried to tell them that I spoke to my doctor and that this was all cleared and it was fine, but they were a little hesitant and, at one point, they said, “Well, why do not you give us the medication and we’ll give it to you?” And I said, “No…” I said, “I wanna keep the medication in my nightstand here so I do not get it mixed up with anything else,” and so they finally said that was fine. *(P22G6PwP)* I wish there was a way for patients who are self - aware to be able to be more self-dosing while they are in the hospital, with some limit per day. They often know their needs better than any staff can. *(P16G4PwP)* …and so a couple times I gave medication to him when he needed to have it and then I just told them that I’d already given it to him. I know they were not happy but we could not wait. It was very hard to get through that this is time sensitive*. (P3G1CP)* I did not trust them [nurses] to give it to him, so I wanted to give him his pills. *(P24G7CP)*
**Desire for flexibility in hospital protocols regarding falls risk and ambulation**	
limitations in mobility protocols and overall ambulation while in the hospital	It was kind of funny that as soon as I said Parkinson’s they put a tag on my hand saying that the fall risk. So after that, they would not let me get off the bed by myself, even though I was pretty able to walk. As long as I’m on the medication, I’m pretty stable. But they put the tag and after that I had to call the nurse every time I want to get off the bed and use the bathroom or anything like that. *(P25G7PwP)* They had fits because I would get out of bed and I’d have to urinate and they would just go ballistic about me getting out of bed but they would not come right away “cause they were dealing with other issues so I took it upon myself to.” So finally at about 6 in the morning they brought a port-a-potty into the room. But you know they should have done that sooner if they did not want me getting out of the bed “cause alarms went off left and right.” *(P7G2PwP)* “You should continue on moving and get out of the bed” that is not something they [healthcare team] discussed at all. *(P19G6PwP)* It was difficult to convince them that I’m pretty capable of getting up and walking by myself. They just would not listen to me. And I did not make a fight because I knew that they had their protocols and that they are following theirs. And like I said, if my fall risk increases, if I realize that I’m getting weak or I stumble more, I would totally welcome that protocol. I know it is useful. *(P16G4PwP)* To keep someone in bed even two days, my husband has had to relearn to walk twice during the length of his hospitalizations. And it could be prevented if you were able to get out of bed and walk around the room or down the hall every day and it’s just not encouraged. They come and do the physical therapy evaluation before discharge. And they should be getting them out of bed every day. It’s so quick that you lose the ability to walk. *(P14G3CP)* And they would not let him out [to be discharged] because they said his balance was so bad. But he had been in bed for 3 days. So of course, his balance is bad. That’s a long time to be in a hospital where he’s totally in bed. He could not get up and walk. He just had to be in that bed. *(P10G3CP)* [for inpatient PT evaluation] he was able to walk across the room and down the corridor. So there wasn’t an issue that time. But then when he eventually started doing it at home in a home setting, it was obvious that maybe it was not possible to do. If he would go outdoors, walk down the driveway, and get into the vehicle or drive somewhere and come back, then he could not get out of the vehicle. He would just collapse on the ground*.(P1G1CP)* For Parkinson’s, every patient is different and their needs are different. So when I said, “You know, he needs help getting up out of the bed.” And he has to have help getting out of a chair even now, and so I had to tell them that because they would come into the room and say, “Okay, it’s time to go to the bathroom,” and they would take the pole or let him take the pole. He cannot do that, you know? His disability does not allow for that*… (P24G7CP)* Even if not walking but at least just some kind of sitting down exercises, and to take him to have some kind of activities. We really do not have that. It has not been happening in a hospital on its own, is not it? It should happen especially with Parkinson’s patients*.(P21G6CP)*
**The need for disability—informed hospital environment**	
Preservation of independence in the hospital environment	Sometimes it was difficult, especially the dinner, to cut the food up I mean, ‘cause my hands start shaking. *(P19G6PwP)* My husband would always try to order finger food that he would rather feed himself and he always had difficulty with that hand coordination after surgery for several days. And so to think about maybe adding items that are finger foods, do not fall apart when you pick them up. *(P14G3CP)* You do not give a Parkinson’s person with tremor a full glass of water because it’ll be all over the place. You also offer them a straw and always do a half glass of water. Little things like that make all the difference. *(P24G7CP)*
Desire for accommodations for PD—specific care needs	It’s hard for me to sleep so when I get to sleep, I’m not really happy when somebody wakes me up and they would come in and have to take my temperature and my blood pressure. *(P7G2PwP)* Every time I’ve been in the hospital, they keep me fairly close to the nurse’s desk and especially during change in shift, you have got all these nurses and everybody congregating at this one spot and they are all talking at once. It gets kind of noisy especially in the middle of the night when they are changing shifts*. (P8G2PwP)* Nurses did not care that I had Parkinson’s and, therefore, disabled in ways that they were not familiar with. *(P23G7PwP)* It was hard to use urinal. Needless to say, there were times that he would get wet or whatever and I asked [the nurse] if she would help and she said, “No, he needs to learn to just do it himself.” And it kind of threw me off “cause I did not expect that answer.” *(P1G1CP)* The problem is as they get older and sicker, we also get older and sometimes also sicker. It was harder and harder for me to spend those nights in the hospital because those chairs that they give you are very uncomfortable. *(P18G5CP)*

Although none of the participants’ hospitalizations was directly related to PD symptoms, the presence of a PD diagnosis and whether the health care team (HCT) actively acknowledged the PD diagnosis had a significant impact on the perceptions of care of both for the PwPs and CPs. In both planned and unplanned hospitalizations, trust in the HCT was immediately gained when the team openly acknowledged the patient’s diagnosis of PD and demonstrated knowledge about specific considerations during hospital stays, anesthesia, and post-hospitalization rehabilitation.

**1.2. Hospitalization experience differed according to whether hospitalizations were planned or unplanned.**

Overall, the participants’ experiences with planned hospitalization were positive starting from the ability to choose their HCT with previous experience in PD care.

*“That was really important to me. I wanted to make sure that there was somebody that could do a good job with hip replacement but they have had patients that had Parkinson’s, so they knew that that was an element that was different.” (P15G4PwP).*

PwP chose the date of their planned hospitalization to ensure the presence of CP during the hospital stay and after the discharge: *“And so I actually chose that particular surgery time so that I knew my daughter would be around… It was at Christmas time and… She is a teacher, and she was actually off for the next two and a half weeks.” (P5G1PwP).*

During planned hospitalizations, PwPs had support from the rehabilitation services, and were given precise discharge instructions regarding the primary cause of hospitalization. Still, participants admitted to struggling to maintain the timing of their PD medication dosage during the hospital stay and their discharge instructions did not reference their diagnosis of PD.

In contrast, unplanned hospitalizations were described as “*chaotic*,” requiring quick decision-making from either PwPs or CPs on whether an ER visit was warranted. For those with unplanned hospitalizations, not one participant mentioned that they had a plan for contacting their neurologist or primary care physician. PwPs and CPs from multiple focus groups commented on delayed access to PD medications in the ER as well as perceived challenges with care delivery (e.g., HCT ability to perform intravenous cannulation placement or chest X-ray) due to prominent PD symptoms such as tremor. CPs were active participants in the decision to go to the hospital for unplanned hospitalizations, and in some cases, drove PwP to the ER. At the ER and once admitted, CP played an essential role in describing the usual state of health of the PwP and helped communicate any changes in their symptoms from baseline to the HCT.

**2. The presence of PD-specific care needs posed an additional challenge for participants during hospitalization: “I expect them to be aware of the fact that I’m different.”**

Specific needs affecting the experience from admission to discharge were identified, including knowledge of PD medications, proper medication management, improved hospital ambulation protocols, and preservation of independence in the hospital environment.

**2.1. Numerous hurdles with PD medications management lead to dissatisfaction with hospital care among the participants.**

Prior to any type of hospitalization, across all focus groups, PwPs and CPs were concerned about the availability of PD medications and, as a result, packed and brought their medications to the hospital. All participants reported issues with consistent and timely delivery of PD medications at all stages of hospitalization, from admission to discharge. For some, medications were substituted or re-arranged without explanation, which created mistrust toward HCT.

*“They were giving me different looking medications that they assured me was the same thing, just a different manufacturer or whatever it was. Every time they gave me something and I looked at it and questioned it. … it was very disconcerting for me.” (P22G6PwP).*

Most participants reported needing to have continuous discussions about their medication regimens with their care team. Trust in the HCT further eroded when the PwP perceived that the team lacked knowledge of commonly used medications for PD.

*“Everyone had a general understanding of Parkinson’s, but not what I would consider, really, decent depth. And especially when it came to the medication, that was a tangible way of judging [the team]- that was something that had to happen and they needed to understand why it was important.” (P26G7PwP).*

To ensure the correct medications were taken on time and as prescribed, many PwPs and CPs chose to administer their own medications during hospitalization. This was accomplished with or without nursing staff awareness. One CP explained: “*I did not trust them to give it to him, so I wanted to give him his pills.*” *(P24G7CP)* While acknowledging possible limitations, many participants expressed a desire to see protocols around medication self-administration in the hospital. As one participant shared: “*I wish there was a way for patients who are self-aware to be able to be more self-dosing while they are in the hospital, with some limit per day. They often know their needs better than any staff can*.” *(P16G4PwP).*

**2.2. The restrictive nature of the hospital fall prevention protocols, along with dissuasion of ambulation, was discordant to participants needs to maintain mobility in the hospital.**

Participants who experienced planned hospitalizations for orthopedic issues received prompt postoperative physical therapy (PT) with encouragement for daily ambulation. However, during unplanned hospitalizations, PwPs struggled to advocate for their ambulation needs and reported limited or no evaluation by PT and decreased mobility due to bed confinement. While CPs were commonly present at the bedsides of PwP, they were unsuccessful in advocating for more physical activity. In all focus groups, both PwPs and CPs remarked that the immobility of the PwP was not a concern for HCT. While some participants actively advocated for more physical activity and an assessment by a PT during their hospital stay, others did not, but still expressed their concerns during their focus group.

There were multiple PwPs with good postural stability and no history of falls who were deemed to be a “fall risk” during their hospitalization. “*It was kind of funny that as soon as I said ‘Parkinson’s’ they put a tag on my hand saying that I have fall risk. So, after that, they would not let me get off the bed by myself, even though I was able to walk*.” *(P16G4PwP)* The discrepancy between PwP needs to maintain mobility in the hospital, and the restrictive nature of the hospital fall prevention protocol, along with dissuasion of ambulation, was unsettling to the patients. “*They had me in lockdown mode because I was the fall risk… I would just attempt to escape from Alcatraz.*” *(P25G7PwP)* While participants acknowledged fall prevention as an important aspect of hospitalization, not many PwPs mentioned success in their advocacy to the HCT to revert fall prevention protocols despite obvious distress that such protocols created during their hospital stay.

**2.3. Hospital environment was not accommodating toward participants’ existing motor and non-motor limitations, indicating the need for disability-informed hospital environment.**

Both CP and PwP participants reported feeling that PwP’s sense of independence was significantly altered in the hospital. They described the impact of poor fine motor control (due to bradykinesia or tremor) on PwP’s ability to attend to daily tasks, such as eating and preferring finger foods on the menu, drinking from half-filled glasses to prevent spillage, and requiring assistance with managing urinals or pushing buttons on bed controls. Both PwPs and HCT preferred CPs to be at the bedside to aid in communication related to PD (e.g., low volume of voice, cognitive issues), although many CPs commented on the lack of accommodations for them, including limited space at the bedside or uncomfortable chairs. Some participants described significantly interrupted night sleep due to vital signs assessments, hearing conversations at the nursing station, or being awakened early to take morning medications. Some CPs observed that sleep interruptions created subsequent confusion and delirium and negatively affected the hospital experience for PwPs. Notwithstanding the reason for hospitalization, when accommodations for PD-specific care needs were included in hospital care, the experience was perceived by PwPs and CPs as more positive than when accommodations were excluded.

## Discussion

Our study used an innovative approach to define care needs of PwPs and CPs in the inpatient setting. By gathering first-hand experiences from direct stakeholders, we used qualitative and patient-centered methods to define the challenges and opportunities for improving hospitalization for PD. Thematic analysis revealed unique needs of PwPs and CPs while in the hospital, including the desire for individualized treatment plans and approaches, and the impact of the PD diagnosis on the perception of care during hospitalization.

Consistent with previous literature, the timely provision of PD medications was a key factor in the experience of and satisfaction with care for participants (Barber et al., 2001; Burroughs et al., 2007; Gerlach et al., 2011). In a systematic review examining the prevalence of adverse events related to medication errors, 31% of PwPs expressed dissatisfaction in the way their PD was managed (Gerlach et al., 2011). A more recent study focusing on motor outcomes identified medication errors as the most important factor in motor deterioration during hospitalization (Gerlach et al., 2013). Owing to challenges with medications in the hospital, most participants in our study proceeded with or desired medication self–management. Studies in other patient populations demonstrated the benefits of carefully applying validated medication self–administration protocols during hospitalization and after discharge (Manias et al., 2006; Vanwesemael et al., 2018a,b). The potential benefits and barriers to PD medication self-administration have been explored in outpatient settings; however, no study to date has assessed attitudes toward inpatient medication self-management in the PD population (Tuijt et al., 2020; Armstrong et al., 2021). Strategic and evidence-based medication self-management protocols for PwPs in the early stages or with support of CPs could empower PwPs and CPs and alleviate the workload on hospital staff.

Participants highlighted an important opportunity to improve PCC through individualized assessment of fall risk and flexibility in fall prevention protocols. To our knowledge, the study of falls and fall prevention protocols in hospitalized PwPs does not exist, even though gait and balance deficits were found in 41% of hospitalized PD patients, and prospective studies documented falls in up to 70% of PwPs (Wood et al., 2002; Bernhard et al., 2018). In older adults, a multidisciplinary and patient-centered approach to the development and implementation of hospital fall prevention protocols has been beneficial and could serve as a roadmap for similar quality improvement initiatives for PwPs (Covinsky et al., 2011; Hempel et al., 2013; Matarese et al., 2015). Participants in our study also strongly advocated for safe mobilization and early assessment by rehabilitation therapists during their hospital stay because of their fear or the reality of worsening PD motor symptoms due to immobility. Although there is a lack of literature on the safety and feasibility of early mobilization for hospitalized PwPs on general wards, studies show the benefits of early mobilization after surgery in PD (Macaulay et al., 2010; Schroeder et al., 2015). Walking during hospitalization is effective for older adults, promoting mobility, shortening hospital stays, and increasing likelihood of discharge to home (Hastings et al., 2018). Interventions to encourage mobility in this population show promise in preventing hospital-associated functional decline and maintaining prehospitalization mobility (Wassar Kirk et al., 2018; Cohen et al., 2019; Resnick and Boltz, 2019). Reported barriers to physical activity during hospitalization include insufficient staffing to assist with or encourage mobility, illness symptoms, fear of falls, and a discouraging hospital environment (Brown et al., 2007; Boltz et al., 2011; Koenders et al., 2020). Our study participants alluded to similar barriers to mobilization during their hospital stays. In addition to further research on fall prevention protocols for hospitalized PwPs, identifying patients with low fall risk and encouraging safe ambulation could be the first step to translate the well-established benefits of sustained mobility from outpatient to inpatient care for PD and to empower PwPs and CPs during hospitalization (Ellis et al., 2021).

In our study, nearly two-thirds of participants lived with the PD diagnosis for more than 6 years, and 84% experienced unplanned hospitalizations, emphasizing the complexity of care in the mid- and later stages of PD. The participants’ descriptions of challenges with navigating the hospital environment, including but not limited to tremor preventing the ease of intravenous cannulation placement, difficulty picking up and swallowing food that was served, and using hospital equipment like nurse call buttons, were not anticipated by the researchers when this study was designed. These PD-related challenges point to the hidden impact of hospitalization on one’s sense of independence. In addition, many CPs mentioned worsening of cognitive function or the development of delirium in PwPs while hospitalized. Our study methods precluded us from identifying specific practices implemented for delirium prevention; however, participants in multiple focus groups mentioned poor sleep protection for PwP during hospitalization. This was similar for CPs, who left the hospital feeling exhausted from reportedly sitting in uncomfortable chairs, monitoring and speaking for their PwP, and continuing to care for their partners once discharged home. Patients diagnosed with PD are fivefold more likely to be treated for delirium than patients from the general population, which may be related to non-motor symptoms in PD, such as dementia, cognitive impairment, and sleep disturbances (Figueroa-Ramos et al., 2009; Stavitsky et al., 2012; Lubomski et al., 2015). Since hospitalization places older adults and PwPs alike at risk for new or worsening disability and reduces likelihood of recovery, several successful interventions have been employed to modify hospital environment and improve patient experience and outcomes (Covinsky et al., 2011; Cohen et al., 2019; Resnick and Boltz, 2019; de Foubert et al., 2021). The hospital environment has a significant impact on patient satisfaction with care, and thus, it could be beneficial to develop and adopt customized hospital accommodations for PwPs to optimize outcomes and decrease risk of complications (Skelly et al., 2014; Rapport et al., 2019).

When patients with chronic conditions are admitted to the hospital, they are expected to switch from being the leader of their own care to being a passive consumer who resumes self-management only upon discharge. Consequently, during hospitalization, the combined stress of acute and chronic illness, set against the background of ongoing pressure to advocate for their unique needs, may be all-consuming for PwPs and CPs. Yet, this can be easily overlooked by HCTs as they are focused on medical management of the acute condition that caused hospitalization. In our study, PwPs and CPs sought active acknowledgement of PD diagnosis by their HCT and adjustment of the hospital communications, protocols or even environment, all of which underscore the impact of PD diagnosis on their perceptions of and experiences with inpatient care. Chronic care advocates argue that hospitals will continue to play a key role in chronic disease care, despite how many acute hospitalizations can be avoided, as most chronic conditions are characterized by acute exacerbations requiring admission (Hernandez et al., 2009; De Regge et al., 2017). Innovative care delivery models, such as the Chronic Care Model, recognize the importance of better preparing hospitals for a role in chronic illness management and demonstrate positive outcomes associated with specialized knowledge of PD among inpatient HCTs (Skelly et al., 2015; Siu et al., 2017). Thus, key findings from our study support acknowledging and accommodating the intersectional needs between the chronic condition of PD and the acute reason for hospitalization of the PwP.

The strength of our study is the use of purposeful sampling, a technique widely used in qualitative research, to identify and select information-rich cases for the most effective use of limited resources (Patton, 2002). Purposive sampling allowed us to identify and select individuals in different stages of PD and ensure that we would capture maximum variation of hospitalization experiences. Qualitative analysis can reveal themes in the data that otherwise may be difficult to identify using quantitative approaches. Focus groups, as a qualitative method, carried an additional strength by creating information—rich data. Focus groups allowed people to discuss the relevant topics with other PwPs and CPs using their own language, to build upon each other’s accounts and promoted “memory synergy,” bringing forth a “collective memory” of varied perspectives on similar experiences during hospitalization (Kamberelis and Dimitriadis, 2013). One of the limitations of our study is that we were unable to recruit CPs who experienced planned hospitalizations with their PwP, and, as a result, this perspective was not represented in our focus groups. Our sample was largely white, despite having intentionally expanded our recruitment efforts to include PwPs and CPs from diverse demographic backgrounds. Racial and ethnic differences in diagnosis, care experiences, and treatment utilization with PD are well known (Ben-Joseph et al., 2020). Therefore, the findings from this study likely cannot be generalized to the overall PD population and must be further validated in people with varied racial, ethnic, socioeconomic, and clinical backgrounds. Because the focus groups occurred months after their hospital stays, participants’ reports were subject to recall bias, and their nonclinical knowledge may have restricted their abilities to identify all factors impacting their hospitalizations. Despite the fact that some of the focus group participants were hospitalized during the COVID-19 pandemic, the discussion did not elucidate robust comments to draw any conclusions about the effect of the COVID-19 pandemic on their experience with hospital care. Despite these limitations, this study provided a novel opportunity for PwPs and CPs to describe their own realities of their hospitalization experiences.

Our study adds to the canon of literature on hospital care for PD. Still, several concepts brought forth by this study warrant further exploration. There is an opportunity to further investigate the role and impact of advocacy by PwPs and CPs on healthcare delivery, as well as explore methodology to capture the real-time experiences of PwPs and CPs during hospitalization, as has been accomplished in other medical conditions (Gualandi et al., 2021). Additionally, the methods and findings of this study serve as good starting points for understanding the hospital experiences of those with atypical parkinsonian syndromes, including progressive supranuclear palsy and multiple system atrophy, given the complexity of symptoms, rapid disease progression, profound lack of awareness of these rarer neurodegenerative diagnoses within the medical community, and the current dearth of research on hospital care for atypical parkinsonism (Dayal et al., 2017; O’shea et al., 2023).

## Conclusion

Our qualitative study draws attention to the significant impact a PD diagnosis can have on planned and unplanned hospital stays, even when the reason for care is not directly related to PD. It highlights the plethora of unique needs PwPs and their CPs have during hospitalization. Findings from this study can be used to inform patient-centered interventions aimed at improving the experience with hospital care for PD, including tools that help PwPs prepare for and advocate during hospitalization as well as ensuring flexibility, as appropriate, within hospital protocols. Empowering PwPs and CPs to communicate their questions, concerns, goals, and needs, both generally and regarding PD, with HCT in the hospital setting, thus applying the principles of PCC, could lead to the care they desire and set them up for higher likelihood of positive outcomes following hospitalization.

## Data availability statement

The raw data supporting the conclusions of this article will be made available by the authors, without undue reservation.

## Ethics statement

The studies involving humans were approved by the Office of Human Research Ethics the University of North Carolina at Chapel Hill. The studies were conducted in accordance with the local legislation and institutional requirements. The ethics committee/institutional review board waived the requirement of written informed consent for participation from the participants or the participants’ legal guardians/next of kin because the research was deemed to have no more than minimal risk of harm to subjects and involves no procedures for which written consent is normally required outside of the research context (e.g., many phone or mail surveys, “man in the street” interviews, etc.).

## Author contributions

NB: Conceptualization, Data curation, Formal analysis, Funding acquisition, Investigation, Methodology, Project administration, Resources, Software, Visualization, Writing – original draft, Supervision, Validation. JS: Conceptualization, Data curation, Funding acquisition, Investigation, Project administration, Resources, Methodology, Writing – original draft. SG: Data curation, Methodology, Software, Writing – review & editing, Formal analysis, Validation. PM: Conceptualization, Data curation, Methodology, Writing – review & editing, Supervision.

## Funding

The study was supported by Parkinson’s Foundation Community Outreach Resource Education grant PF-CORE-2008 and Parkinson’s Foundation Center of Excellence Award PF-COE-929858.
