# Supplementary material for: More than medications: a patient-centered assessment of Parkinson’s disease care needs during hospitalization
Source: Front Aging Neurosci. 2023 Sep 28;15:1255428. doi: 10.3389/fnagi.2023.1255428 (PMC10569176; doi:10.3389/fnagi.2023.1255428)
Supplement: Supplementary file 1 [file Table_1.pdf]

Appendix 1. Focus group discussion guide.

Topics	Questions	Prompts	Probes
**Before hospitalization**			
Introduction	Please introduce yourself	How long ago was your hospitalization?	
Before Hospital	What made you decide to go to the hospital?	Was it a planned visit? (e.g., scheduled surgery)<br>Was it an unexpected situation? (e.g., change in functioning, fall)	Before coming to the hospital (either for planned or unplanned admission), how did you prepare for the hospitalization?<br>Did you call your neurologist and they suggested you go to the hospital? Call PCP? Went to Urgent care? Call 911? Had plan on what to do?
**In the Hospital**			
Patient and team interaction	What is one thing you feel went well during your hospital experience?	Can you tell me more about that?	
	To what degree do you feel that the medical team met your needs?	Can you tell me more about that?	

	How do you feel your diagnosis of Parkinson's disease impacted how you were treated, if at all?	Were you treated differently because of Parkinson's? In what ways?	Has anybody else had a similar experience? Did anybody have a different experience?
	What was your impression of how knowledgeable your treatment team was of Parkinson's disease?	Do you have an example?	Has anybody else had a similar experience? Did anybody have a different experience?
	To what extent were you involved in medical/care decisions?		Does anybody else have anything different to add?
	To what extent was your family member or care provider involved in medical/care decisions?	Did medical providers involve both you and your family member/care provider in decisions? Please elaborate.<br>Did medical providers communicate differently or the same with you as they did with your family member/care provider? Please elaborate.	Has anybody else had a similar experience? Did anybody have a different experience?
Medications and PD	While in the hospital, were you given your Parkinson's medications as prescribed, on time?	If not, please elaborate.	Did you need to advocate for yourself/partner to get your medications on time?

	While in the hospital, were your Parkinson's medications substituted or adjusted by the team?	If yes, please elaborate.	Did you need to advocate for yourself/partner to get your medications on time?<br>Did you need to bring your own medications to use in the hospital?<br>Did you use your own medications?
Rehab service and PD	Did you need rehabilitation or PT/OT evaluation while in the hospital?	If yes, was it provided? How soon?	If you are using a cane or a walker on regular basis at home, were those provided for you in the hospital?<br>Were you encouraged to ambulate in the hospital?
Hospital environment	How did you find your environment in the hospital?	Were you able to attend your activities (bathroom, grooming, toileting)? Did you need help?<br>Do you think any of your PD symptoms were impacted by the hospital environment?	If you needed help, who provided it?<br>Has anybody else had a similar experience?<br>Did anybody have a different experience?
**Advocacy and empowerment in the hospital**			

Aware in Care kit	Do you have an Aware in Care kit and if so, did you bring it with you?	If yes, how were you able to utilize the contents during the hospitalization?	
Advance care directives	Have you had any advance care directive paperwork prior to hospitalization?	Have you brought any advance care paperwork in?<br>Who, if anybody, reviewed it?	
Advice for future hospitalization/ to other patients	Thinking on your experiences that you shared today, are there ways your hospitalization, in regard to your Parkinson's care, could have been made better?	What advice would you give someone with Parkinson's who needs to be hospitalized?	
	Anything else that comes to mind that we did not talk about in our discussion?	Tell me more about that	
